# Address of Prof. Harriss

**Published:** 1871-12

**Authors:** 


### ADDRESS OF PROF. JURIAH HARRISS, M. D.

---

We have been much gratified in reading the Introductory Address of Prof. Juriah Harriss, delivered before the Class of the Savannah Medical College. It affords us a real pleasure to note the genuine professional tone of this address, so hearty is its annunciation of correct moral principles, and withal, so modest in its uterances in everything concerning the college and faculty, with which the speaker is connected.

We have the highest esteem for the noble professional men of Savannah, among whom many are identified with the Savannah Medical College; and the address of Prof. Harriss cannot fail to win for that institution warm-hearted friends in every portion of the country.

The future of the profession, in a large measure, lies in the cultivation of the *moral* attributes of the students committed to the care of physicians by fond parents. We should make them gentlemen as well as doctors, and in proportion as we succeed in accomplishing the first, so will we the other—and in elevating the dignity of the profession.

No parent, unless he desires his son to become a thief, would subject him to the pernicious example and maxims of the notorious swindlers of "Peter Funk" shops. Such a step would result in the inevitable ruin of the son, and be a calamity to the country and his friends. In medical training and education, example has much to do with the future of the medical student. If his preceptor is pure in thought and word—a man of unswerving integrity and truth, these great principles will be impressed upon him for life—and a gentleman and a scholar will be begotten under the hallowed influence of such a mind and heart.

But the moral training does not stop here. Many instructors in our colleges, loose sight of the moral dignity they should impart as the highest stimulus to intellectual effort, in their teachings and general deportment. They should teach by example as well as by precept. The bright jewel of truth should sparkle and glow in their words and character; and the pure, spotless integrity of the Christian gentleman should pervade the lecture-room, no less than the private walks of life.

In speaking thus, we have had reference to the faculty of the Savannah Medical College. We do not desire or intend to underrate other institutions in their moral and medical teachings, but we speak of our friends of Savannah, warranted by a personal acquaintance and friendship. All over our country no more of honor and high professional integrity can be found—no more cultivated and gifted minds in medical lore—than in Savannah.

The address of Dr. Harriss is but a rivulet streaming from the great heart-fountain of such men—true to principle and devoted to science.

Dr. Harriss, in alluding to the high educational status of the Savannah Medical College, says :

" Our requirements of students, and our elevated status of medical education, are in concert with the ethics of the profession of the country, and sustained by the oldest and best Colleges of the land, in our State controversy, in ignoring irregular schools."

No higher eulogy could be spoken in praise of the institution he represents, than the one uttered above—" in concert with the ethics of the profession of the country, and sustained by the oldest and best colleges of the land, in our State controversy, in ignoring irregular schools."

All honor to Savannah, for its noble stand in this controversy—a controversy begun and still being waged by parties antagonistic to the ethics and the high standard of medical education. All honor to Savannah for its fearless, out-spoken repudiation of irregular schools and their manipulators. Not a spot—not a stain rests upon her proud escutcheon to tarnish her honor or blacken her reputation.

